# A Uniform Funnel Array for DOA Estimation in FANET Using Fibonacci Sampling

**DOI:** 10.3390/s25092651

**Published:** 2025-04-22

**Authors:** Siwei Huo, Ming Zhang, Yongxi Liu, Shitao Zhu

**Affiliations:** School of Information and Communications Engineering, Xi’an Jiaotong University, Xi’an 710049, China; siweihuo@stu.xjtu.edu.cn (S.H.); liuyongxi@stu.xjtu.edu.cn (Y.L.); shitaozhu@xjtu.edu.cn (S.Z.)

**Keywords:** direction-of-arrival (DOA) estimation, correlative interferometer, Fibonacci sampling, phase differences, similarity function

## Abstract

The Flying Ad-Hoc Network (FANET) is an important component of the 6G communication system. In order to achieve the precise positioning of unmanned aerial vehicle (UAV) nodes in a FANET when satellite navigation signals are unavailable, simple and accurate direction-of-arrival (DOA) estimation methods are required. In this paper, we propose an improved correlative interferometer method to estimate the DOAs of the UAVs in a FANET. This method adopts a uniform funnel array (UFA) configuration, which consists of a uniform circular array (UCA) and an additional element located above the center. This configuration improves the estimation accuracy for DOAs with large polar angles because it utilizes the degree of freedom in the vertical aperture. In addition, the Fibonacci sampling strategy is employed to overcome the polar clustering phenomenon exhibited by latitude–longitude sampling. Furthermore, in the interferometer, only partial phase differences are used to reduce the storage burden. When calculating the similarity function, we adopt the triangular function instead of the cosine function to improve computational efficiency. The simulation results show that the proposed UFA improves the DOA estimation accuracy by 65.56% over the planar UCA for signals with large polar angles. Moreover, Fibonacci sampling improves the DOA estimation accuracy by 11.54% as compared to the latitude–longitude sampling.

## 1. Introduction

In recent years, unmanned aerial vehicle (UAV) technology has made remarkable progress, from single UAV operations to multi-UAV collaborations, with an increasingly wide range of application scenarios [1,2,3,4]. Notably, a network consisting of multiple UAVs is called the Flying Ad-Hoc Network (FANET), which is a key component of the 6G communication system [5]. Accurate relative position information among UAVs is crucial to maintaining the stability and efficiency of the network [6,7]. Conventional localization methods through GPS or Beidou navigation systems [8] become unavailable when satellite signals are weak due to electromagnetic interfering and jamming, particularly in military applications [9]. In this case, direction-of-arrival (DOA) estimation becomes critical for the maintenance and reconfiguration of UAV formations [10].

Many DOA estimation methods have been developed in the past several years, including monopulse beamforming [11], spatial spectrum estimation [12] and interferometer direction finding [13], which are widely used in airborne platforms. The monopulse beamforming method has high DOA estimation accuracy; however, it is sensitive to environmental factors and has high system complexity. The spatial spectrum estimation methods, such as Multiple Signal Classification (MUSIC) [14,15] and the Estimation of Signal Parameters via Rotational Invariance Technique (ESPRIT) [16,17] algorithms, are able to estimate multiple signals simultaneously with high accuracy. However, their computational complexity is very high due to the covariance matrix estimation, eigenvalue decomposition, and spectral peak search, making it difficult for them to meet the real-time processing requirement in a FANET. In comparison, the interferometer method is a more suitable technique to be applied in an airborne environment because of the advantages of simple implementation, low complexity, and high accuracy [13,18].

Mutual coupling can reduce the accuracy of DOA estimation if it is not handled properly, especially for arrays with a compact structure. Fortunately, this problem can be solved via calibrations. In refs. [19,20], an online calibration method was proposed and improved to enhance the performance of MUSIC by treating mutual coupling as nuisance parameters. In ref. [21], an offline calibration of the array was performed to obtain more accurate steering vectors used in DOA estimation.

There are two types of interferometers for DOA estimation—the phase interferometer [22] and the correlative interferometer (CI) [23]. The former suffers from the problem of phase ambiguity. Although correct direction measurement can be achieved using ambiguity resolution techniques [24], these methods have many special requirements regarding the array configuration. Moreover, it is difficult to obtain accurate measurement results at low signal-to-noise ratios (SNRs). The correlative interferometer estimates the DOA by comparing the measured phase differences across multiple antennas with those stored in the dictionary. It uses correlation processing to weaken the adverse effects on direction measurement due to mutual coupling and other hardware impairments. Despite of its simplicity, it can be applied flexibly to arbitrary array structures [25].

The most common form of array configuration used in the correlative interferometer is the uniform circular array (UCA) [26,27]. In ref. [28], it was shown that, when computing the loss functions, the method based on the cosine function performed better than the method based on the correlation coefficient. In ref. [29], a direction-finding method based on the UCA of directional sensors and an extra central omnidirectional sensor was proposed to estimate multiple coherent or incoherent sources, thereby improving the speed and accuracy of the estimator. However, planar arrays were used in the previous methods, which have a low vertical resolution and a low estimation accuracy at large polar angles. Due to the limited space and dynamic flying attitudes of UAVs, it is necessary to design a simple array configuration, as well as a high DOA estimation accuracy method [30].

In this paper, we design a simple 3D array structure named uniform funnel array (UFA) by adding an additional element above the center of the UCA to improve the vertical resolution. Moreover, when constructing the candidate angle grids of the correlative interferometer, the traditional latitude–longitude sampling suffers from polar clustering phenomenon, i.e., there are redundant points near the pole of the upper hemisphere. In order to overcome this problem, we construct the candidate angle grids using the Fibonacci sampling method [31,32], which generates points that are more uniformly distributed on the upper hemisphere. Another important issue with the interferometer is the baseline selection. For an array consisting of *M* elements, any two array elements are able to form a baseline. Each baseline can output a phase difference measurement. If all baselines are used, there will be M(M−1)/2 values to be stored for each direction. To reduce the data storage burden and the computational complexity, we examine the impact of different baseline selection strategies on the performance of DOA estimation. It is shown that only 2(M−1) baselines can achieve high accuracy. In addition, when calculating the similarity function, the triangular function is adopted instead of the cosine function, which further improves the computational efficiency. Since we are using a rather simple array, the problem of mutual coupling can be overcome via the offline calibration method.

The key contributions of this paper are summarized as follows:

1. We design a simple UFA to improve the DOA estimation accuracy of correlative interferometer for signals coming from large polar angles.
2. We propose a candidate angle grid generation method based on Fibonacci sampling, which overcomes the polar clustering phenomenon exhibited by the latitude–longitude sampling.
3. We employ the partial baselines method to construct the phase difference dictionary, which saves storage space and improves computational efficiency.
4. We use the triangular function rather than the cosine function to calculate the similarity function, which reduces the computational cost.

Simulation results show that, at large polar angles with SNR =10 dB, the UFA improves estimation accuracy by 65.56% over the planar UCA. Moreover, the use of Fibonacci sampling improves the estimation accuracy by 11.54% as compared to latitude–longitude sampling.

The rest of this paper is organized as follows: Section 2 explains the principle of the correlative interferometer and the improvement in the similarity function. Section 3 describes the details of the proposed method, including the array structure design, sampling strategy, and baseline selection. Simulation experiments are given in Section 4 to validate the effectiveness of the proposed method. Section 5 includes the discussion and conclusions.

## 2. Principle of Correlative Interferometer

The correlative interferometer is a direction-finding equipment that correlates the measured phase difference with the phase difference dictionary. The direction in the dictionary corresponding to the maximum correlation coefficient is chosen as the estimated DOA.

Let A be the candidate angle output set of the correlative interferometer, as follows:

(1) $$A≜\left\{\left(\theta_{1},\varphi_{1}\right),\left(\theta_{2},\varphi_{2}\right),\cdots ,\left(\theta_{N},\varphi_{N}\right)\right\},$$

where $\left(\theta_{n},\varphi_{n}\right)$ denotes the candidate polar and azimuth angle, and *N* is the cardinality of the set A.

**Figure 1:**
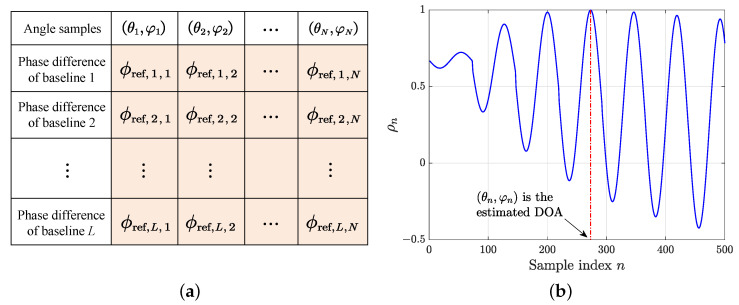
Phase difference dictionary diagram and the variation curve of $\rho_{n}$. (**a**) Phase difference dictionary diagram. (**b**) The variation curve of $\rho_{n}$.

The phase difference dictionary is shown in Figure 1a, denoted by $\Phi_{\mathrm{ref}}\in R^{L\times N}$, where *L* is the number of baselines. Assume that a signal is incident from an unknown direction, and the phase difference vector calculated by the measured phase is $\phi_{\mathrm{mea}}\in R^{L}$. The regular similarity function based on the correlation operation is as follows [33]:

(2) $$\rho_{n}=\frac{\phi_{\mathrm{mea}}^{T}\phi_{\mathrm{ref},n}}{∥\phi_{\mathrm{mea}}∥∥\phi_{\mathrm{ref}}∥}\;,$$

where $\phi_{\mathrm{ref},n}$ is the *n*-th column of the phase difference dictionary $\Phi_{\mathrm{ref}}$, and $∥\cdot ∥$ stands for the 2-norm. The variation curve of $\rho_{n}$ is shown in Figure 1b. The angle $\left(\theta_{n},\varphi_{n}\right)$ in A corresponding to the maximum $\rho_{n}$ is the estimated DOA of the incoming signal.

Equation (2) cannot accurately discriminate the angle due to the 2π phase ambiguity. To overcome this problem, the phase differences are expressed in complex vector form, denoted as $\phi_{\mathrm{mea},c}={\left[e^{j\phi_{\mathrm{mea},1}},\cdots ,e^{j\phi_{\mathrm{mea},L}}\right]}^{T}$ and $\phi_{\mathrm{ref},c,n}={\left[e^{j\phi_{\mathrm{ref},1,n}},\cdots ,e^{j\phi_{\mathrm{ref},L,n}}\right]}^{T}$. Based on the principle of least squares, the DOA can be estimated by minimizing the following function:

(3) $$\begin{array}{cc} J_{n} & ={∥\phi_{\mathrm{mea},c}-\phi_{\mathrm{ref},c,n}∥}^{2}\\ \; & =2\sum_{l=1}^{L}\left[1-\cos\left(\Delta \phi_{l}\right)\right],\;n=1,2,\cdots ,N, \end{array}$$

where $\Delta \phi_{l}=\phi_{\mathrm{mea},l}-\phi_{\mathrm{ref},l,n}$. Therefore, the estimated DOA can be obtained by finding the maximum value of the function $\chi_{n}$ through the following equation [13]:

(4) $$\chi_{n}=\sum_{l=1}^{L}\cos\left(\Delta \phi_{l}\right),\;n=1,2,\cdots ,N.$$

However, implementing the cosine function in the Field Programmable Gate Array (FPGA) usually relies on specific algorithms, such as the coordinate rotation digital computer (CORDIC) algorithm. The complexity and cost of this algorithm is high. Therefore, in order to improve computational efficiency, we calculate the similarity function using the following triangular function:

(5) $$\mathrm{tri}\left(\Delta \phi_{l}\right)=\left\{\begin{array}{cc} -\frac{2}{\pi }\left|\Delta \phi_{l}\right|+1, & |\Delta \phi_{l}|\leq \pi ,\\ \frac{2}{\pi }\left|\Delta \phi_{l}\right|-3, & \pi <|\Delta \phi_{l}|\leq 2\pi . \end{array}\right.$$

**Figure 2:**
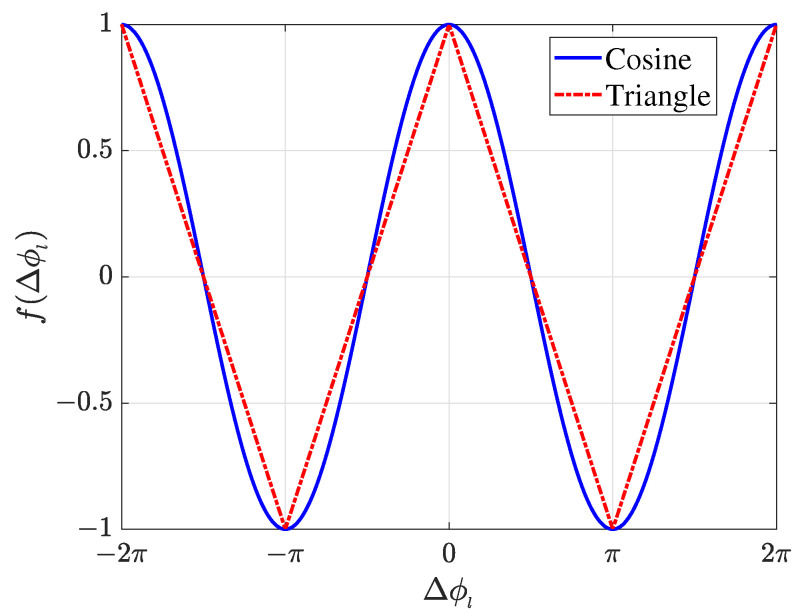
Improved methods for calculating the similarity function.

This function only performs simple absolute value operation. Figure 2 illustrates the difference between triangular and cosine functions. Simulation experiments show that they have almost the same DOA estimation performance. Then, the final similarity function $\chi_{n}^{\prime }$ is given by the following:

(6) $$\chi_{n}^{\prime }=\sum_{l=1}^{L}\mathrm{tri}(\Delta \phi_{l}),\;n=1,2,\cdots ,N.$$

It should be noted that the phase measured by the phase discriminator is in the range [−π,π). To ensure the accuracy of calculating the similarity function, the values in the phase difference dictionary should be restricted to the same range.

Three major factors that strongly affect the DOA estimation accuracy of the correlative interferometer are listed as follows:

1. Array structure;
2. methods for constructing phase difference dictionary;
3. baseline selection.

In the following sections, we explain how these factors can be revised to enhance the performance of the correlative interferometer.

## 3. The Proposed Method: Correlative Interferometer Using Fibonacci Sampling (CIFS)

### 3.1. Array Configuration

**Figure 3:**
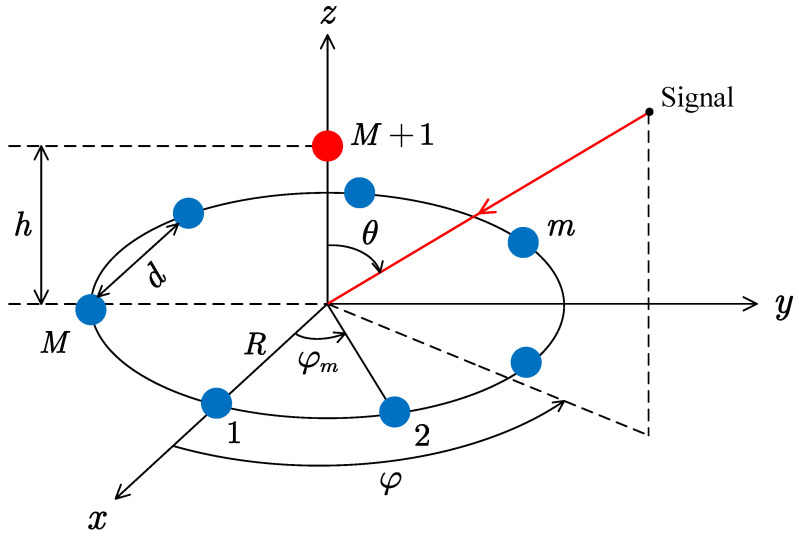
The proposed UFA for DOA estimation.

The conventional correlative interferometer generally utilizes the UCA for DOA estimation. However, it has low estimation accuracy at large polar angles. In view of this problem, we propose a UFA structure as shown in Figure 3. The UFA consists of an *M*-element UCA and an extra element with height *h* located above the center of the UCA.

Suppose there is a signal incident on the UFA with DOA (θ,φ). Then, the signal received by the UFA is given by the following:

(7) x(k)=s(k)a(θ,φ)+n(k),

where $a\left(\theta ,\varphi \right)\in C^{M+1}$ is the steering vector, $n\left(k\right)∼CN\left(0,\sigma^{2}I_{M+1}\right)$ is the noise vector. We premise that signal and noise are statistically independent.

The position coordinate of the *m*-th element in the UCA is as follows:

(8) $$p_{m}={\left[R\cos\left(\varphi_{m}\right),\;\;R\sin\left(\varphi_{m}\right),\;\;0\right]}^{T},\;m=1,2,\cdots ,M.$$

The wavenumber vector $\beta \in R^{3}$ [34] is as follows:

(9) $$\beta =-\beta_{0}{\left[\sin(\theta )\cos(\varphi ),\;\;\sin(\theta )\sin(\varphi ),\;\;\cos(\theta )\right]}^{T},$$

where $\beta_{0}=2\pi /\lambda$, with λ being the wavelength. The inner product of Equations (8) and (9) is as follows:

(10) $$\begin{array}{cc} \beta^{T}p_{m} & =-\beta_{0}R\left[\sin\left(\theta \right)\cos\left(\varphi \right)\cos\left(\varphi_{m}\right)+\sin\left(\theta \right)\sin\left(\varphi \right)\sin\left(\varphi_{m}\right)\right]\\ \; & =-\beta_{0}R\sin\left(\theta \right)\cos\left(\varphi -\varphi_{m}\right). \end{array}$$

Alternatively, the coordinate of the element located above the center of the UCA is as follows:

(11) $$p_{M+1}={\left[0,\;\;0,\;\;h\right]}^{T}.$$

Then, the steering vector of the UFA is the following:

(12) $$a\left(\theta ,\varphi \right)=\left[\begin{array}{c} e^{-j\beta^{T}p_{1}}\\ e^{-j\beta^{T}p_{2}}\\ \vdots \\ e^{-j\beta^{T}p_{M}}\\ e^{-j\beta^{T}p_{M+1}} \end{array}\right]=\left[\begin{array}{c} e^{j\beta_{0}R\sin\left(\theta \right)\cos\left(\varphi -\varphi_{1}\right)}\\ e^{j\beta_{0}R\sin\left(\theta \right)\cos\left(\varphi -\varphi_{2}\right)}\\ \vdots \\ e^{j\beta_{0}R\sin\left(\theta \right)\cos\left(\varphi -\varphi_{M}\right)}\\ e^{j\beta_{0}h\cos\left(\theta \right)} \end{array}\right].$$

When the additional vertical element is employed, the effective area of the array at larger polar angles would be enlarged compared to the planar UCA. From the perspective of the wavenumber vector β, this additional element utilizes the $\beta_{z}=\cos\left(\theta \right)$ degree of freedom in the estimation algorithm.

### 3.2. Fibonacci Sampling

**Figure 4:**
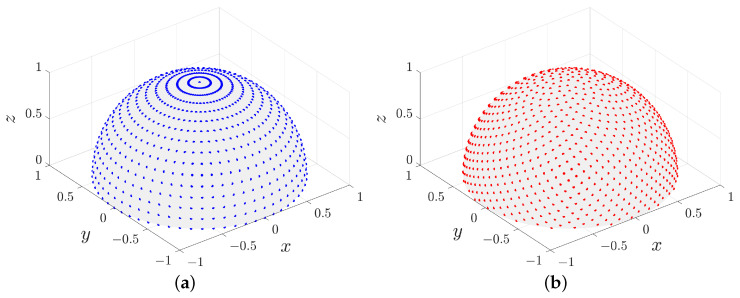
Two sampling methods with the same number of samples. (**a**) Latitude-longitude sampling. (**b**) Fibonacci sampling.

When constructing the phase difference dictionary, the angular samples are critical in the correlative interferometer. However, the traditional latitude–longitude sampling suffers from the polar clustering phenomenon, as shown in Figure 4a. It can be seen that the points are very dense near the north pole and sparse near the equator. To address this problem, we adopt Fibonacci sampling to generate more uniformly distributed samples on the sphere.

The Fibonacci sequence was introduced by the mathematician Leonardo Fibonacci, who used the example of rabbits reproducing, thus it is also known as the “rabbit sequence”. The sequence is a linear recursive sequence starting with the third term, where each term is equal to the sum of the previous two terms, i.e., $a_{n+2}=a_{n+1}+a_{n}$. Its first few terms are 1,1,2,3,5,8,13,⋯. The general formula of the sequence is as follows:

(13) $$a_{n}=\frac{1}{\sqrt{5}}\left[r^{n}-\frac{{\left(-1\right)}^{n}}{r^{n}}\right],\;n\in N,$$

where

(14) $$r=\frac{1+\sqrt{5}}{2}\approx 1.618,$$

and $r^{-1}\approx 0.618$ is the golden ratio. Substituting the Fibonacci sequence into the cylindrical equal product projection formula, the coordinates (in radians) of the *n*-th Fibonacci sampling point on the upper hemisphere can be obtained using the following equation [35]:

(15) $$\theta_{n}=\frac{\pi }{2}-\mathrm{arc}\sin\left(\frac{n}{N_{\mathrm{fib}}}\right),$$

(16) $$\varphi_{n}=\mathrm{mod}\left(n,r\right)\times 2\pi r^{-1},$$

where $N_{\mathrm{fib}}$ is the number of Fibonacci sampling points. The points generated by the Equations (15) and (16) form the set $S=\left\{\left.\left(\theta_{n},\varphi_{n}\right)\right|n=0,1,\cdots ,N_{\mathrm{fib}}-1\right\}$, which gives a more uniform distribution on the upper hemisphere as illustrated in Figure 4b.

The following two critical properties of Fibonacci spherical sampling need to be emphasized:

1. Only one sampling point exists on each latitude line.
2. The longitudinal spin between two sequential points along the generated spiral is the golden angle α, i.e.,
  (17) $$\alpha =360^{\circ }\left(1-r^{-1}\right)\approx 137.52^{\circ }.$$

### 3.3. Baseline Selection

**Figure 5:**
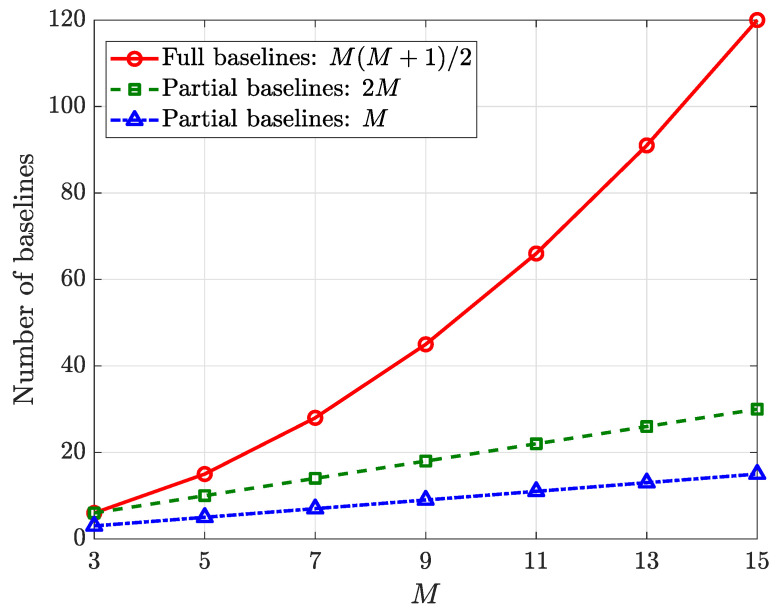
Number of baselines using different selection strategies.

Any two elements in Figure 3 can form a baseline to compute their phase differences, and there are M(M+1)/2 baselines in total. However, if all the baselines are used for estimations, the storage space in the hardware is wasted and the estimation performs many redundant calculations. Therefore, only partial baselines are required in practical applications. For example, the long baselines of the UCA can be selected in combination with the baselines formed between the upper element and the UCA (2M in total), or the baselines formed by the upper element and the UCA (*M* in total). When *M* is odd, the number of baselines given by these three baseline selection methods is shown in Figure 5. It can be seen that when *M* is large, a huge space can be saved if the latter strategies are used.

**Figure 6:**
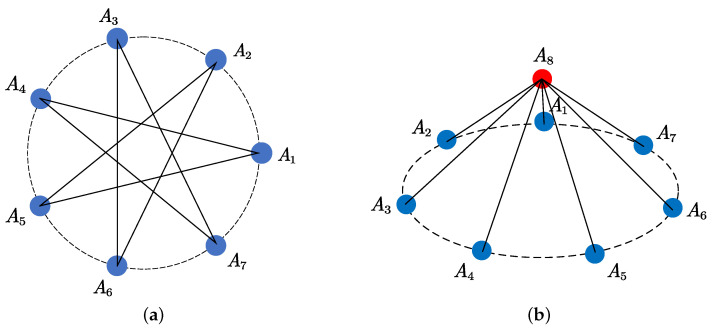
Baseline selection method for DOA estimation. (**a**) Long baselines of UCA. (**b**) Baselines formed by the center element and the UCA.

In this paper, considering the implementation and accuracy issues, the second method is chosen in Figure 5, i.e., 2M baselines. When M=7, the selected baselines are shown in Figure 6, which include seven long baselines of the UCA and seven baselines formed by the center antenna and the UCA. This method saves half of the storage space and dramatically improves the computational speed compared to storing all baselines.

From Equation (12), when the candidate angle is $\left(\theta_{n},\varphi_{n}\right)$, the phase difference between the $m_{1}$-th element and the $m_{2}$-th element of the UCA in Figure 6a is as follows:

(18) $$\phi_{\mathrm{ref},m,n}=\beta_{0}R\sin\left(\theta_{n}\right)\left[\cos\left(\varphi_{n}-\varphi_{m_{1}}\right)-\cos\left(\varphi_{n}-\varphi_{m_{2}}\right)\right],\;m=1,2,\cdots ,M.$$

The phase difference between the *m*-th element of the UCA and the element above the UCA in Figure 6b is as follows:

(19) $$\phi_{\mathrm{ref},p,n}=\beta_{0}\left[R\sin\left(\theta_{n}\right)\cos\left(\varphi_{n}-\varphi_{m}\right)-h\cos\left(\theta_{n}\right)\right],\;p=M+1,M+2,\cdots ,2M.$$

Equations (18) and (19) constitute the *n*-th column of the phase difference dictionary $\Phi_{\mathrm{ref}}$. Note that, in practice, the phase differences need to be calibrated due to the presence of mutual coupling and manufacturing issues.

## 4. Numerical Experiments and Results Analysis

### 4.1. Experiment 1: Array Structure

In the first experiment, the array structure shown in Figure 3 is adopted, where the radius of the 7-element UCA located in xy-plane is R=0.58λ, the spacing between adjacent elements is d=0.5λ. The height of the center element is h=0.8λ.

In the FANET, the polar angles of the impinging signals are in the range of $\left[60^{\circ },90^{\circ }\right]$, and the azimuth angles are in the range of $\left[0^{\circ },360^{\circ }\right)$. There are a total of L=32,760 sampling points in the latitude–longitude lattice corresponding to the angular interval of $\Delta \theta =\Delta \varphi =1^{\circ }$. The number of Monte-Carlo simulations is $N_{\mathrm{sim}}=5000$. The simulations are conducted on a personal computer with one 12th Gen Intel(R) Core(TM) i7-12700 2.10 GHz CPU.

In this paper, we use the 3D error metric calculated as follows [36]:

(20) $$\gamma =\mathrm{arc}\cos\left[v^{T}\left(\theta_{e},\varphi_{e}\right)v\left(\theta_{t},\varphi_{t}\right)\right],$$

where the error between the true DOA $\left(\theta_{t},\varphi_{t}\right)$ and the estimated DOA $\left(\theta_{e},\varphi_{e}\right)$ is measured, and $v\left(\theta ,\varphi \right)=\beta /\beta_{0}$ is the direction vector of (θ,φ). The root-mean-square error (RMSE) is as follows:

(21) $$\mathrm{RMSE}=\sqrt{\frac{1}{N_{\mathrm{sim}}}\sum_{n=1}^{N_{\mathrm{sim}}}\gamma_{n}^{2}}.$$

**Figure 7:**
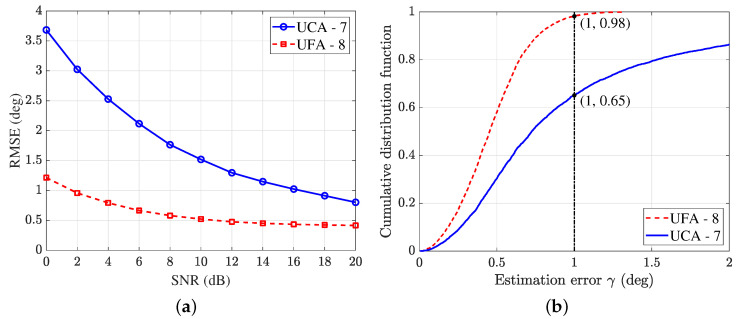
Comparison of the proposed 8-element UFA and the 7-element UCA. (**a**) RMSE curves with K=100. (**b**) CDF curves with SNR =10 dB, K=100.

In the experiment, the number of snapshots is fixed at K=100. When SNR varies from 0 dB to 20 dB, the RMSE of the 8-element UFA and the 7-element UCA is shown in Figure 7a.

It can be seen that the proposed array structure has a significant improvement in estimation accuracy, which shows a greater improvement in the low SNR region and a smaller improvement in the high SNR region. In Figure 7b, the cumulative distribution function (CDF) of the estimation error γ with SNR =10 dB is plotted. It can be seen that the probability of $\gamma \leq 1^{\circ }$ is 65% with the UCA. However, the probability of $\gamma \leq 1^{\circ }$ is 98% when the UFA is used. In this case, the RMSE given by the UCA is $1.51^{\circ }$ while that of the UFA is $0.52^{\circ }$. The estimation accuracy is improved by 65.56%.

**Figure 8:**
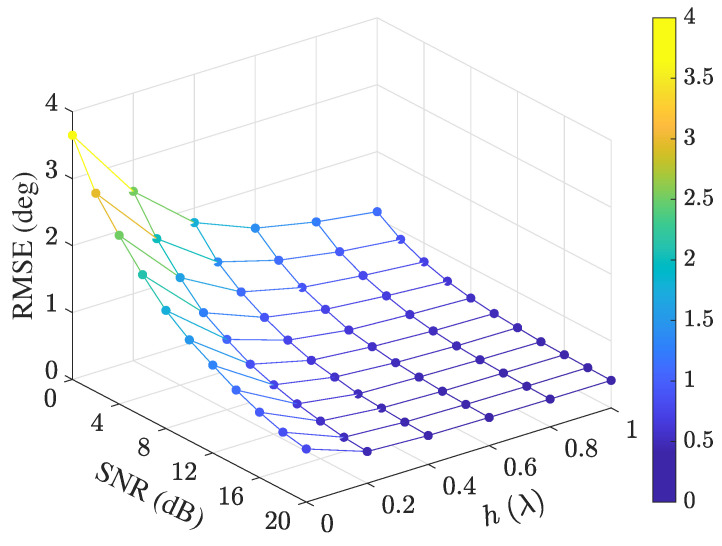
Variation of RMSE with SNR and *h* under K=100.

When both the SNR and *h* vary, the variation of RMSE using the UFA is shown in Figure 8. It can be seen that the estimation accuracy increases along with SNR and *h*.

### 4.2. Experiment 2: Fibonacci Sampling Method

In the second experiment, the performances of Fibonacci sampling and the latitude–longitude sampling are compared under the same number of samples, i.e., $N_{\mathrm{fib}}=N_{\mathrm{ll}}=32,760$. In the simulation, the polar angles of the impinging signals are in the range of $\left[60^{\circ },90^{\circ }\right]$, and the azimuth angles are in the range of $\left[0^{\circ },360^{\circ }\right)$.

**Figure 9:**
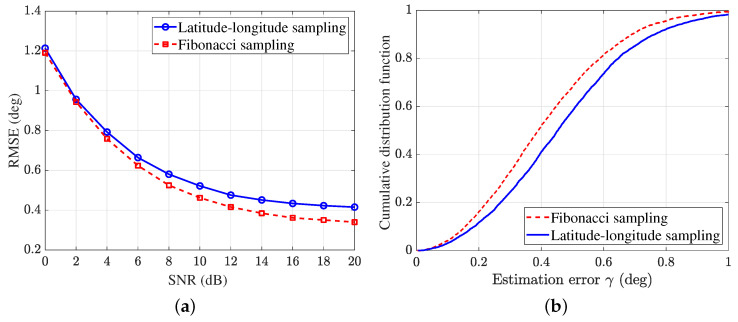
Comparison of the latitude–longitude sampling and Fibonacci sampling. (**a**) RMSE using different sampling methods with K=100. (**b**) CDF curves with SNR =10 dB, K=100.

Figure 9 shows the simulation results with two sampling strategies. It can be seen that Fibonacci sampling has a higher estimation accuracy than the latitude–longitude sampling, particularly in the high SNR region. When SNR =10 dB, the RMSE of the latitude–longitude sampling is $0.52^{\circ }$ and that of Fibonacci sampling is $0.46^{\circ }$; as a result, the DOA estimation accuracy is increased by 11.54%. This performance improvement is due to the more uniform sample distribution, which aligns with our previous analysis.

**Figure 10:**
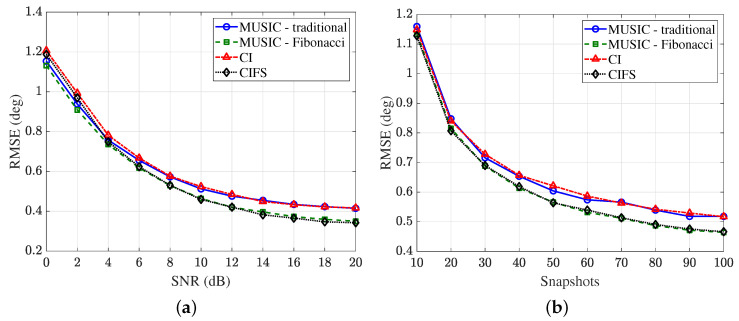
Comparison of CI algorithm and MUSIC algorithm. (**a**) RMSE versus SNR with K=100. (**b**) RMSE versus snapshots *K* with SNR =10 dB.

The performance of MUSIC and CI using the different sampling strategies are shown in Figure 10, where 3000 independent simulation trials are performed. As can be seen from the figure, the accuracy of both algorithms is almost the same using the identical sampling strategy. Moreover, the accuracy of both algorithms is improved under Fibonacci sampling.

**Figure 11:**
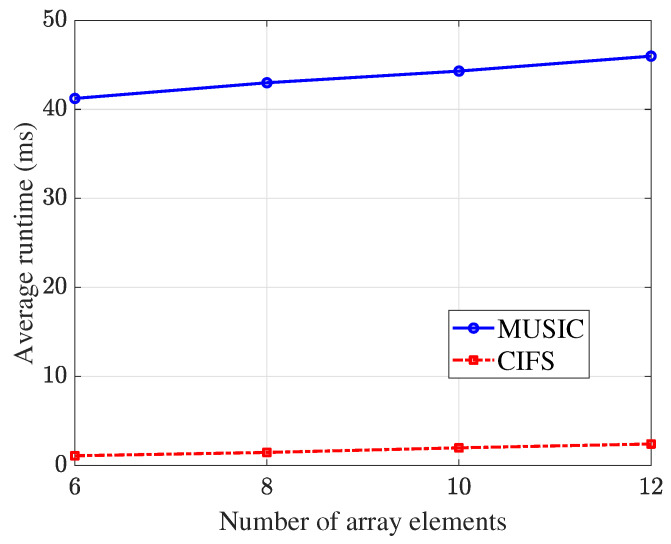
Curves of the runtime for estimating one DOA with SNR =10 dB, K=100.

The average runtime of the MUSIC and CI algorithms using different array elements is shown in Figure 11, where 1000 independent trials are performed in total. It can be seen that under the same parameter settings, the CI algorithm runs much faster than the MUSIC algorithm.

It should be noted that the CI method can only discriminate one impinging signal at one frequency point. However, communication channels in FANET are separated by frequency bins and/or time slots. Therefore, the proposed method can estimate the DOAs of multiple UAVs.

### 4.3. Experiment 3: Baseline Selection

In the third experiment, we examine the impact of different baseline selection strategies on the estimation accuracy. The parameters are the same as in Section 4.1.

**Figure 12:**
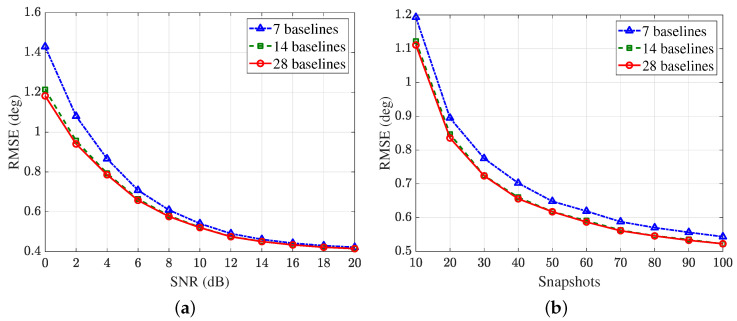
RMSE of three baseline selection strategies. (**a**) RMSE versus SNR with K=100. (**b**) RMSE versus snapshots *K* with SNR =10 dB.

The simulation results are shown in Figure 12. It can be seen that the DOA estimation accuracy is the lowest when only seven baselines are selected. At low SNRs, the accuracy of storing 14 baselines is slightly lower than that of the full baselines (28 in total). While at high SNRs, the accuracy of this two methods are almost identical. However, when all baselines are used in the experiment, the runtime is 35.58 s, while the runtime corresponding to 14 baselines is only 10.12 s, showing a significant increase in computational efficiency with a marginal loss on accuracy.

## 5. Conclusions and Discussion

In this paper, we proposed an improved correlative interferometer DOA estimation method. Four factors were exploited to improve the estimation performance, including array structure, phase difference dictionary construction, baseline selection, and similarity function. Specifically, the proposed UFA structure improved DOA estimation accuracy at large polar angles owing to the enhanced vertical resolution. Furthermore, Fibonacci sampling was used to construct the candidate angle grids, which led to a more uniform distribution of samples as compared to the traditional latitude–longitude lattice. In addition, when constructing the phase difference dictionary, only partial baselines were used to improve the computational efficiency with a marginal loss on the accuracy. In addition, when calculating the similarity function, we adopted the triangular function instead of the cosine function to increase the computational speed.

In practical applications, one advantage of the proposed method was its fast running speed, which naturally fits the moving scenarios in UAVs. Indeed, this method was implemented in an FPGA, in which the parameters are shown in Table 1. It can be seen that the UAV could only move a very small distance during the measurement interval. Therefore, the dynamic movements of the UAVs did not affect the estimation accuracy. Moreover, the proposed DOA estimation method primarily relied on phase differences, which did not need to demodulate the impinging signal. Consequently, the synchronization and connection stability of the communication link were not the main concerns in this method. However, they could play an important role when range measurements is required in FANETs.

## Figures and Tables

**Table 1:** Parameters of the proposed algorithm implemented in the FPGA.

Sampling Frequency (MHz)	Number of Sample Points	Measurement Time (μs)	UAV Speed (km/h)	Distance Traveled by UAV (mm)
100	100∼1000	1∼10	200∼300	≤0.83

## Data Availability

Data are contained within the article.
